# Editorial: Cancer-Associated Defects in the DNA Damage Response: Drivers for Malignant Transformation and Potential Therapeutic Targets

**DOI:** 10.3389/fgene.2015.00355

**Published:** 2015-12-22

**Authors:** Marcel A. T. M. van Vugt, H. Christian Reinhardt

**Affiliations:** ^1^Department of Medical Oncology, University Medical Center Groningen, University of GroningenGroningen, Netherlands; ^2^Department I of Internal Medicine, University Hospital CologneGermany; ^3^Cologne Graduate School of Ageing Research, University of CologneCologne, Germany

**Keywords:** checkpoint blockade, cell cycle, DNA repair, synthetic lethality, DNA damage response (DDR), rewiring

Transformation of normal cells into cancer cells almost invariably goes along with increased levels of DNA damage. An important source of DNA damage is the enhanced activity of growth-promoting transcription factors, such as *MYC*. Oncogenic activation of these transcription factors aberrantly stimulates DNA replication, which leads to replication stress and ensuing DNA breaks. Cells respond to this type of stress by activation of the DNA damage response (DDR). The DDR is a complex signaling network, displaying multiple levels of cross-talk and feed-back control. Its kinase-driven signaling axes ensure rapid responses to DNA lesions, which is complemented by its transcriptional axis that warrants maintained signaling. Ultimately, activation of the DDR prevents further proliferation and thus provides time to repair genotoxic lesions, and in case of excessive levels of DNA damage promotes permanent cell cycle exit (senescence) or programmed cell death (apoptosis). Activation of the DDR thus prevents the outgrowth of incipient tumor cells, early during tumorigenesis.

In line with the ability to eliminate damaged cells from the proliferative compartment, the DDR clearly has a tumor-suppressive role. Indeed, many tumor cells have inactivated parts of the DDR, which allows proliferation in the context of DNA damage-inducing oncogenes. Not only does partial inactivation of the DDR allow growth of transformed cells, it also provides opportunities for therapeutic intervention. Loss of specific DDR components leaves tumor cells more dependent on their remaining DDR components, especially under conditions of elevated levels of DNA damage induced by chemo/radiotherapy. Identification of such synthetic vulnerabilities may lead to more targeted therapies, in which therapeutic inactivation of the DNA damage response in cancer cells will create more potent anti-cancer strategies.

DNA damage and the repair thereof are increasingly recognized as key pathways in normal physiology, as well as being pathways defective in multiple pathological conditions, including accelerated aging, neurodegeneration and cancer. In addition, the fundamental research into the molecular underpinning of DNA repair that was initiated more than 50 years ago has now translated into drugs that inactivate key components of the DDR with high levels of specificity. Noteworthy, PARP inhibitors, the first molecularly targeted anti-cancer drugs that exploit the DNA repair defect present in *BRCA1*- or *BRCA2*-mutant cancers were FDA-approved at the end of 2014. The increasing importance of this field is illustrated by the 2015 Albert Lasker Awards for Biomedical Research being awarded to Stephen Elledge and Evelyn Witkin, pioneers in uncovering the cellular response to DNA damage. In addition, the 2015 Nobel prize for chemistry was awarded to Thomas Lindahl, Paul Modrich, and Aziz Sancar for their seminal work on DNA repair.

In this “Research Topic” entitled: “Cancer-associated defects in the DNA damage response: drivers for malignant transformation and potential therapeutic targets,” 10 papers have been published, focusing on various aspects of DNA damage signaling, its effects on cellular viability and its use in cancer therapy.

Increasingly, we realize that the DDR is complex. Rather than being a cell-intrinsic kinase-driven linear pathway, we understand that cell–cell communication is involved, and that it encompasses multiple different post-translational protein marks. Brinkmann et al., describe how ubiquitin signaling plays a central role in the DNA damage response, whereas Jaiswal and Lindqvist describe how extracellular signaling is used to communicate the presence of damaged DNA to neighboring cells. Von Morgen et al. and Lezzi and Fanciluuli, describe the R2TP complex and Che-1/AATF, respectively, as novel components of the cellular response to DNA damage. Ohms et al. describe how retrotransposons are at the basis of increased genomic instability and Shatneyeva et al. portray the interplay between the DNA damage response and escape from immune surveillance.

Torgovnick and Schumacher describe how defects in DNA repair contribute to cancer initiation, and conversely, create opportunities for targeted treatment of those cancers. As an example of these therapeutic consequences, Knittel et al., report how *ATM* loss in chronic lymphocytic leukemia creates synthetic lethal interactions with inactivation of certain DNA repair pathways. Additionally, Syljuasen et al., describe how therapeutic inactivation of DNA damage checkpoint kinases could be exploited in the treatment of lung cancer, whereas Krajewska and van Vugt review how modulation of DNA repair through homologous recombination can be utilized as a therapeutic strategy.

Combined, the papers in this Research Topic underscore the complexity of the cellular response to DNA damage, and highlight how mechanistic insight into the (re)wiring of DDR signaling in cancer cells can be exploited to develop novel cancer therapeutics.

## Conflict of interest statement

The authors declare that the research was conducted in the absence of any commercial or financial relationships that could be construed as a potential conflict of interest.

